# Editorial: Purinergic Pharmacology

**DOI:** 10.3389/fphar.2019.00021

**Published:** 2019-01-23

**Authors:** Francisco Ciruela, Kenneth A. Jacobson

**Affiliations:** ^1^Unitat de Farmacologia, Departament Patologia i Terapèutica Experimental, Facultat de Medicina, IDIBELL, L'Hospitalet de Llobregat, Universitat de Barcelona, Barcelona, Spain; ^2^Institut de Neurociències, Universitat de Barcelona, Barcelona, Spain; ^3^Molecular Recognition Section, Laboratory of Bioorganic Chemistry, National Institute of Diabetes and Digestive and Kidney Diseases, National Institutes of Health, Bethesda, MD, United States

**Keywords:** adenosine, G protein-coupled purinergic receptors, ATP, purinergic signalling, adenosine transport, caffeine, purinergic pathophysiology, ligand-gated purinergic ion channels

The purine nucleotides and nucleosides constitute important extracellular signaling molecules acting as neurotransmitters and neuromodulators. Indeed, extracellular adenosine 5′-triphosphate (ATP) and adenosine, tightly controlled by nucleotidases, ribokinases, deaminases, and transporters, signal through a rich array of purinergic receptors. These receptors, which emerged early in evolution, are among the most abundant in living organisms controlling many physiological actions, thus becoming promising therapeutic targets in a wide range of pathological conditions. Thus, while P1 receptors are selective for adenosine, a breakdown product of ATP, P2 receptors are activated by purine nucleotides, as well as P2Y receptors being activated by pyrimidine nucleotides. Interestingly, purinergic receptors, both G protein-coupled (i.e., P1 and P2Y) and ligand-gated ion channel (i.e., P2X) receptors, are involved in many neuronal and non-neuronal mechanisms, including pain, immune responses, exocrine and endocrine secretion, platelet aggregation, endothelial-mediated vasodilatation and inflammation, among others.

Purinergic receptors are ubiquitously expressed throughout the body, thus compromising the specificity of receptor subtype-selective drugs and increasing the possibility of side effects upon pharmacological intervention. However, the extracellular levels of purines may fluctuate enormously, thus distinct purinergic receptors responding differently to low and high concentrations of endogenous purines are called into action while cells are exposed to multiple purinergic signaling molecules. Therefore, the same cell usually concurrently expresses different subtypes of P1 and P2 receptors, which allows the integration of purinergic transmission into short- and long-term signaling events. Consequently, drug selectivity constitutes another important pharmacological goal within the purinergic field. Indeed, the development of potent and selective synthetic agonists and antagonists for purinergic receptors has been the subject of medicinal chemistry research for decades. In addition, allosteric modulators of purinergic receptors have been successfully developed. Interestingly, these compounds allow the manipulation of the endogenous purinergic system in an event-responsive and temporally specific manner, thus offering a unique therapeutic window when compared to orthosteric compounds. Finally, the functioning of the purinergic system could be also manipulated by modulating the metabolism and/or uptake of extracellular purine nucleotides and nucleosides. Overall, there is no doubt that purinergic pharmacology is growing fast and becoming an attractive field for pharmacotherapeutic development.

In this timely research topic, an overview of the purinergic pharmacology is provided through 61 articles written by 439 authors. This successful compilation contains 15 reviews, 5 mini reviews, 3 hypothesis and theory papers, 1 perspective, and 37 original research papers. The reviews summarize the currently available knowledge on the role of purinergic signaling, focusing on the pathophysiology and its therapeutic potential (Burnstock), for example, in mast cell degranulation and its most relevant disease, asthma (Gao and Jacobson), pulmonary arterial hypertension (Alencar et al.), amyotrophic lateral sclerosis (Sebastião et al.), neurodevelopmental disorders (Fumagalli et al.), epilepsy (Alves et al.), neurological diseases with motor symptoms (Oliveira-Giacomelli et al.), motivational dysfunction and depression (López-Cruz et al.), and inflammatory diseases (Savio et al.), In addition, some of the reviews provide more mechanistic opinions, for instance on purinergic transmission in psychostimulant addiction (Ballesteros-Yáñez et al.), carotid body physiology (Conde et al.), and platelet heterogeneity (Koupenova and Ravid). Also, some hints about the molecular mechanism of ligand binding and activation by adenosine (Carpenter and Lebon) and ATP (Di Virgilio et al.) receptors is provided. Finally, the impact of adenosine transport in purinergic signaling is also reviewed (Pastor-Anglada and Pérez-Torras).

Subsequently, five minireviews highlight diverse aspects of purinergic signaling. For instance, the role of adenosine and its receptors in T cell development in the thymus (Köröskényi et al.) and the regulation of the activity of immune cells and enteric nervous system (Dal Ben et al.). Thus, the most promising purine-based strategies are summarized for the treatment of inflammation-related disorders, including the recent development of nanobodies against key targets of purinergic system (Menzel et al.). Next, the current knowledge on the pathophysiological involvement of purinergic receptors in white and brown adipocytes and their potential use in metabolic disorders is reviewed (Tozzi and Novak). Also, the recent emerging data involving the ATP-gated P2X7 ion channel as a potential drug target for central nervous system disorders including neuropsychiatric conditions (Bhattacharya).

In the section “Hypothesis and Theory” the molecular determinants of small-molecule ligand (i.e., BzATP and ivermectin) binding at P2X receptors is reviewed grounded in structure-based docking studies (Pasqualetto et al.). Next, the interplay of adenosine receptors with muscarinic acetylcholine and neurotrophin receptors in the mammalian neuromuscular junction controlling synapse elimination and neurotransmission release is considered (Tomàs et al.). Also, the formation of adenosine A_2A_ and dopamine D_2_ receptor heterotetramers and adenylate cyclase type 5 complexes in striatopallidal neurons are postulated as an integrative device tuning adenosine and dopamine signaling and therefore behavioral effects of adenosine/dopamine-based ligands (Ferré et al.). Finally, a perspective paper discusses the current limitations and highlights future research directions to achieve adenosine receptor-mediated cardioprotection (Lasley).

The research topic contains a series of original research papers covering important aspects of purinergic pharmacology. Thus, several papers focus on the role of adenosine receptors in cancer cells proliferation (Gessi et al.), morphologically altered hippocampal neurons (Pinheiro et al.), hippocampal slices subjected to oxygen and glucose deprivation (Fusco et al.), instrumental animal learning (Li et al.), and rodent models of movement disorders (Núñez et al.). Indeed, the interest of targeting adenosine receptors is also shown by the design of a non-imaging high throughput approach to screen drugs in native receptors (Arruda et al.) and their detection in human brain using positron emission tomography (PET) ligands (Mishina et al.). In addition, some molecular clues about adenosine receptor function are also given, for instance, the formation of transcellular trimeric complexes involving CD26, adenosine deaminase and adenosine A_2A_ receptor (A_2A_R) (Moreno et al.), the A_2A_R-mediated control of glutamatergic synaptic plasticity in prefrontal cortex interneurons (Kerkhofs et al.) and the adenosine A_2B_ receptor (A_2B_R)-mediated control of epithelial-mesenchymal transition by tuning the cAMP/PKA and MAPK/ERK balance (Giacomelli et al.). Also, it is speculated whether A_2A_R may be useful to sustain contractility in failing human hearts and upon ischemia and reperfusion (Boknik et al.). Finally, the role of caffeine in controlling glutamatergic synaptic transmission in human cortical neurons (Kerkhofs et al.) and its adverse effects in an Alzheimer's disease animal model (Baeta-Corral et al.) are investigated.

The role of the purinergic system in microglia and astrocytic function both *in vitro* and *in vivo* is studied in glaucoma (Rodrigues-Neves et al.), prostaglandin E2 signaling (Paniagua-Herranz et al.), extracellular vesicle-based cell communication (Drago et al.), cell migration (Adzic and Nedeljkovic) and proliferation (Quintas et al.). Similarly, the impact on T cells (Shinohara and Tsukimoto; Soslow et al.) and cytokine-induced killer cell function (Horenstein et al.) is also explored. Interestingly, extracellular signaling by guanine-based purines was explored in cultured cells (Pietrangelo et al.; Zuccarini et al.) and in rodent models of movement disorders (Massari et al.).

The extracellular ATP/adenosine ratio is a key element for immune responses, including post-inflammatory ileitis, as described in this research topic (Vieira et al.). Thus, the tissue-nonspecific alkaline phosphatase enzyme and Pannexin-1 channel seem to play an important physiological role regulating the levels of extracellular ATP (Sebastián-Serrano et al.), which ultimately will activate cell surface P2XRs and P2YRs. In addition, it seem that ATP release, at least in the suprachiasmatic nucleus, is under the control of these two kinds of purinergic receptors (Svobodova et al.). Interestingly, P2XRs are allosterically modulated by trace metals (i.e., zinc) and other drugs (i.e., ivermectin) (Latapiat et al.), which also can form heterotrimeric P2X4/P2X7 receptors (Schneider et al.). Importantly, P2X7R is up-regulated and promotes a fibrogenic phenotype in systemic sclerosis (SSc) fibroblasts, thus becoming a potential therapeutic target in SSc patients (Gentile et al.). Conversely, P2YR expression seems to play a key role in eye physiology. Thus, changes in the P2Y_2_/P2Y_1_ expression ratio correlates well with an increment in the intraocular pressure in an animal model of glaucoma (Fonseca et al.). Also, P2Y_12_R blockade facilitate the clearance of lysosomal waste in retinal pigmented epithelial cells, which is relevant for age-related macular degeneration management (Lu et al.). Furthermore, a clinical study revealed that P2Y_12_R blockade does not contribute to risk of osteoporotic fractures in stroke patients, a common adverse effect of transient ischemic attack treatment (Jørgensen et al.). In a separate context, the P2Y_6_R regulates chemokine (i.e., CXCL10) secretion in mouse intestinal epithelia cells, thus regulating gut homeostasis (Salem et al.).

As a neurotransmitter, ATP is stored in secretory vesicles, a process mediated by the vesicular nucleotide transporter (VNUT). In this research topic it is demonstrated that cerebellar granule cells express functional VNUT and that may be implicated in the initial stages of granule cell development (Menéndez-Méndez et al.). Finally, the issue also reports the generation and characterization of a valuable, new tool to study ectonucleotidase nucleoside triphosphate diphosphohydrolase-8 (NTPDase8) (Pelletier et al.) and the characterization of uridine adenosine tetraphosphate's (Up4A) physiological role in *in vivo* heart failure model, i.e., swine aortic banding (Zhou et al.).

Overall, this research topic provides new insights into the vast physiological roles of purinergic signaling and its structural and mechanistic basis. This field offers enormous possibilities for translation of basic science into novel treatments for chronic and acute diseases, while at the same time it presents a challenge to achieve selectivity of drug action.

## Author Contributions

All authors listed have made a substantial, direct, and intellectual contribution to the work, and approved it for publication.

### Conflict of Interest Statement

The authors declare that the research was conducted in the absence of any commercial or financial relationships that could be construed as a potential conflict of interest.

